# Treatment effect of trauma-focused treatment and/or integrated trauma-focused and personality disorder treatment on brain activation during an emotional face task

**DOI:** 10.1192/j.eurpsy.2024.197

**Published:** 2024-08-27

**Authors:** I. Aarts, C. Vriend, O. A. Van Den Heuvel, K. Thomaes

**Affiliations:** ^1^Sinai Centrum, Amstelveen; ^2^Psychiatry; ^3^Anatomy and Neurosciences, Amsterdam UMC, Vrije Universiteit Amsterdam; ^4^Compulsivity, Impulsivity & Attention Program, Amsterdam Neuroscience, Amsterdam, Netherlands

## Abstract

**Introduction:**

Post-traumatic stress disorder (PTSD) and personality disorders are highly comorbid. There is some evidence that trauma-focused treatment normalises activation in brain areas involved in the fear circuit and regions involved in emotion regulation in people with PTSD. Although we assume that working mechanism of personality disorder treatments relies on improving emotion regulation and associated brain regions, there is as of yet little evidence of neurobiological effects of personality treatment on people with PTSD and comorbid PD.

**Objectives:**

To 1) study the effect of trauma-focused and/or trauma-focused and personality disorder treatment n brain activation in participants with PTSD and comorbid personality disorders and 2) relate change in brain activation to symptom improvement.

**Methods:**

Participants with PTSD and comorbid borderline and/or cluster c personality disorders from the PROSPER-trials (Prediction and Outcome Study for PTSD and personality disorders) were randomized to either trauma-focused treatment (TFT) or TFT with personality disorder treatment (TFT+PT). Brain activation was measured with an emotional face task during functional magnetic resonance imaging scanning before and after treatment. Regions of interest for the analyses were the amygdala, dorsal ACC, insula, ventromedial prefrontal cortex (PFC), ventrolateral PFC and dorsolateral PFC. Bayesian multilevel analyses were conducted to analyze change in brain activation. Clinical measures were clinician-administered PTSD severity, self-rated emotion regulation problems, depression severity and dissociation severity.

**Results:**

We included 42 participants with a pre- and posttreatment scan (24 with TFT, 18 TFT+PT). Analyses on the pre-post data are currently being run and will be presented in April.

**Conclusions:**

This is one of the first studies to conduct functional MRI analyses on treatment in participants with both PTSD and personality disorders.

**Disclosure of Interest:**

None Declared

